# Impact of Wearing on Filtration Performance of Electrostatic Filter Face Masks

**DOI:** 10.3390/ijerph19095032

**Published:** 2022-04-21

**Authors:** Anthony P. Pierlot, David L. J. Alexander, Jürg A. Schütz

**Affiliations:** 1 CSIRO (Commonwealth Scientific and Industrial Research Organisation) Manufacturing, 75 Pigdons Road, Waurn Ponds, Geelong, VIC 3216, Australia; tony.pierlot@csiro.au (A.P.P.); jurg.schutz@csiro.au (J.A.S.); 2 CSIRO (Commonwealth Scientific and Industrial Research Organisation) Data61, 20 Research Way, Clayton, Melbourne, VIC 3168, Australia

**Keywords:** new risk factors, respirator masks, disposable face masks, electrostatic enhancement, COVID-19, wear trial

## Abstract

Certified disposable respirators afford important protection from hazardous aerosols but lose performance as they are worn. This study examines the effect of wear time on filtration efficiency. Disposable respirators were worn by CSIRO staff over a period of 4 weeks in early 2020. Participants wore the respirator masks for given times up to eight hours whilst working in laboratory/office environments. At that time COVID-19 precautions required staff to wear surgical (or other) masks and increase use of hand sanitizer from dispenser stations. Results obtained from a test group of ten individuals without health preconditions show an increasing number of masks failing with wear time, while the remainder continue to perform nearly unaffected for up to 8 h. Some masks were found to retain filtration performance better than others, possibly due to the type of challenge they were subjected to by the wearer. However, the rate and extent of decay are expected to differ between environments since there are many contributing factors and properties of the aerosol challenge cannot be controlled in a live trial. Penetration and variability increased during wear; the longer the wear time, the more deleterious to particle removal, particularly after approximately 2 h of wear. This behavior is captured in a descriptive statistical model based on results from a trial with this test group. The effectiveness of the masks in preventing the penetration of KCl particles was determined before and after wearing, with the analysis focusing on the most penetrating particles in a size range of 0.3–0.5 µm diameter where respirator masks are most vulnerable. The basic elements of the study, including the approach to filter testing and sample sanitization, are broadly applicable. Conclusions also have applicability to typical commercially available single-use respirator masks manufactured from melt blown polypropylene as they are reliant on the same physical principles for particle capture and electrostatic enhancement was comparable for the particle size range used for detection.

## 1. Introduction

**Figure 1:**
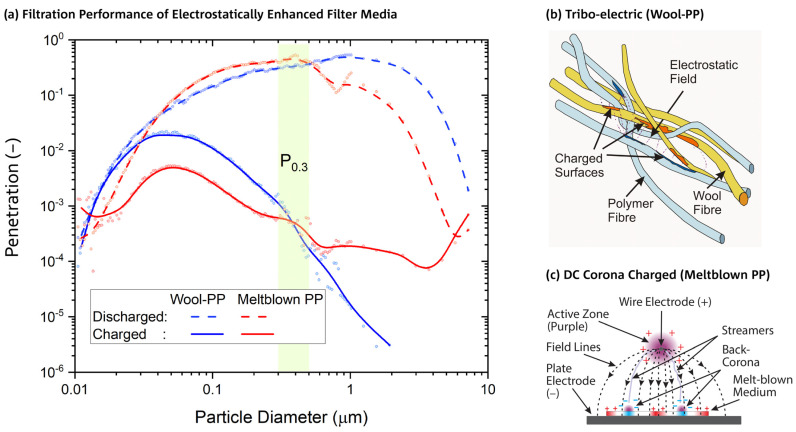
(**a**) An example of the particle-size dependence of penetration for charged and discharged wool/polypropylene (Wool–PP, WP) and melt blown polypropylene (Meltblown PP, MBP; Shanghai Da Sheng [5]) mask media when tested at a face velocity of 0.15 m/s. Pressure drop, thickness, fabric areal density, maximum penetration, and most penetrable particle size (MPPS) of MBP: 200 Pa, 3.0 mm, 280 g/m^2^, 0.5%, 48 nm; WP: 50 Pa, 8.8 mm, 600 g/m^2^, 2.2%, and 46 nm, respectively. The particle diameter size range used for measuring penetration P_0.3_ has been highlighted in green. Trendlines (B-Spline) have been added for illustration purposes to measurements represented by dots. (**b**) Fiber-polymer specific charge distribution in electrostatically enhanced media of the tribo-electric type. (**c**) Charge distribution resulting from DC corona charging using a unipolar electrode configuration (example for unipolar, positive electrode configuration [8]).

The start of the COVID-19 pandemic led to mask supply shortages in clinical settings, particularly those with a tight facial seal (respirators or fitted face masks) and filtration efficiency greater than ~95% (e.g., N95 or P2), leading to rationing and efforts at sterilization and reuse [1,2,3,4]. These types of disposable respirators predominately have a particle capture layer manufactured from melt blown polypropylene (MBP) with electrostatic charge induced from a corona discharge during manufacture. They achieve high levels of particle capture in the aerosol size range of 0.05–2 µm diameter, primarily due to electrostatic fields, with far superior collection efficiency to that of the same type of media in a discharged state (Figure 1). When the electrostatic charge of a filter medium is lost, e.g., through particle capture during use, or exposure to high concentration of alcoholic vapors [5], the medium becomes less effective in preventing the penetration of these sized particles. The penetration is the ratio of the particle concentration measured downstream to that measured upstream of the filter medium. This electrostatic enhancement is necessary to minimize particle penetration under the typical constraints imposed on face mask design such as size, weight, and breathability. The breathability or flow resistance is often given as the pressure drop (Δp) at a flow rate or face velocity consistent with typical breathing rates.

Filter media with an electrostatic charge may also be produced from two types of fibers of different material compositions using tribo-electric charging [6], for example, wool and polypropylene [7]. These nonwoven filter media have substantially reduced flow resistance due to a more open pore structure and a stronger electrostatic effect, resulting in improved breathability and comfort, but at the expense of higher areal density (also known as “basis weight”), increased thickness, and a penetration that is more susceptible to increase due to loss of electrostatic charge. Respirator masks made from low pressure drop filter media are also less prone to leakage through the facial seal. An example of a charged and discharged filter medium manufactured from wool and polypropylene (WP) is shown in Figure 1, together with pertinent filtration performances of a P2-rated, disposable mask medium (Shanghai Da Sheng [5]) made from melt blown polypropylene (MBP) for comparison. The WP pressure drop is 50 Pa, significantly lower than the MBP pressure drop of 200 Pa, while the penetration is higher for particles < 400 nm diameter and lower above. The effect of charge loss, which is represented by differences between fully charged (solid lines) and discharged (dashed lines), is more pronounced for WP above 500 nm particle size. A hypothesis for the reason why the observed electrostatic enhancement is stronger for tribo-electric media in comparison to corona charged media can be based on differences in charge distribution morphology, depicted in sketches of Figure 1b,c: in WP, the charges are segregated according to fiber polymer and, crucially, well intermingled within the nonwoven structure [6]. Charges in MBP, on the other hand, are arranged in relatively large zones of predominantly positive or negative polarity [8], which leads to a less uniform electrostatic field.

Due to severe supply shortages of conventional, melt blown polypropylene respirators during the early phase of the pandemic, WP-type materials were reassessed as potential alternatives. Previous research has demonstrated their effectiveness as respirators when tested according to procedures typically required for certification under various international standards (e.g., NIOSH [9,10], AS1716 [11], and ISO 17420-2 [12]). However, for both types of filter media, there is limited research on degradation of effectiveness due to wear and wear time. To address this limitation, a wear trial of an electrostatic wool/polypropylene filter medium was undertaken. A notable study (published after commencing our study) was a wear trial of an N95 respirator involving 50 health care workers wearing the masks up to 14 days (8 h/day). It demonstrated that mean penetration increased linearly with wear time at the rate of 1.2% per day of wear time, starting from a penetration of 2.2% [13]. Another study investigated the effects of wear in terms of total inward leakage (TIL) that takes the effects of facial seal into account [14]. The N95 rated respirator masks maintained a protection factor above or equal to 10 (which is equivalent to a penetration of 1/10 = 10%, or a filtration efficiency of 90%) for up to 19 wear/uses, over a five-day period. The evaluation was conducted under a daily 7-step “general respirator protection factor” (GRPF) test and supplemented on days 1, 3, and 5 with continuous monitoring of the protection factor in a 12-step protocol referred to as a “simulated workplace protection factor” (SWPF) test. The day-to-day GRPF results were variable but showed overall a noticeable performance deterioration with wear.

This investigation aims to quantify how much and how fast the level of protection afforded by disposable respirators deteriorates during wear in a specific live user setting. The WP filter medium was selected for this study over commercial respirators as the latter were difficult to acquire due to supply shortages and would also require whole masks to be mounted with perfect perimeter seals (e.g., using bees’ wax [15]) for testing both before and after wearing to enable paired comparisons. Whilst the two filter materials have different compositions and structures, they both rely on the principle of small particle capture through electrostatic charge [16], and so the results from wool/polypropylene are also applicable for charged, melt blown filter materials [6,17,18].

## 2. Materials and Methods

### 2.1. Approach

Ten volunteers without health preconditions were selected from organizational staff and they wore masks of a WP filter medium in laboratory/office environments for 1-, 2-, 4-, and 8-h duration during May–June 2020. During these wear periods, volunteers could remove the mask for eating and drinking or if they felt uncomfortable or needed a break. The only requirement was that the masks were worn for the specified period. The performance of each sample was tested before and after wearing to identify any potential reduction in penetration due to wear time. At the time the wear trial was conducted, there were a small number of COVID-19 community transmission cases within Australia. To mitigate potential exposure to the virus, the filter discs were sanitized by a heat treatment at 70 °C for one hour [19] before testing, followed by the same treatment after wearing and then testing again. Eight additional samples that were sanitized (twice) but not worn were also tested as control samples. This study (2020_018_LR and 2021_107_LR) was approved by CSIRO Health and Medical Human Research Ethics Committee.

### 2.2. Filter Materials

The nonwoven WP filter material was produced in November 2004 by blending wool and polypropylene (Table 1), carding, and needle punching. After production the fabric was stored in a laboratory environment wrapped in plastic and had its electrostatic charge regenerated by needle punching in August 2016. This filter medium was chosen for the wear trials as it would easily seal against the wearer’s face due to long-distance electrostatic charge and its penetration was highly stable over time [20]. By comparing filter test results conducted before the trial with historic data, it could be determined that the penetration had approximately doubled due to some loss of charge capacity from storage over 4 years and, therefore, represented the lower limit of fabric performance for these types of filter media.

**Figure 2:**
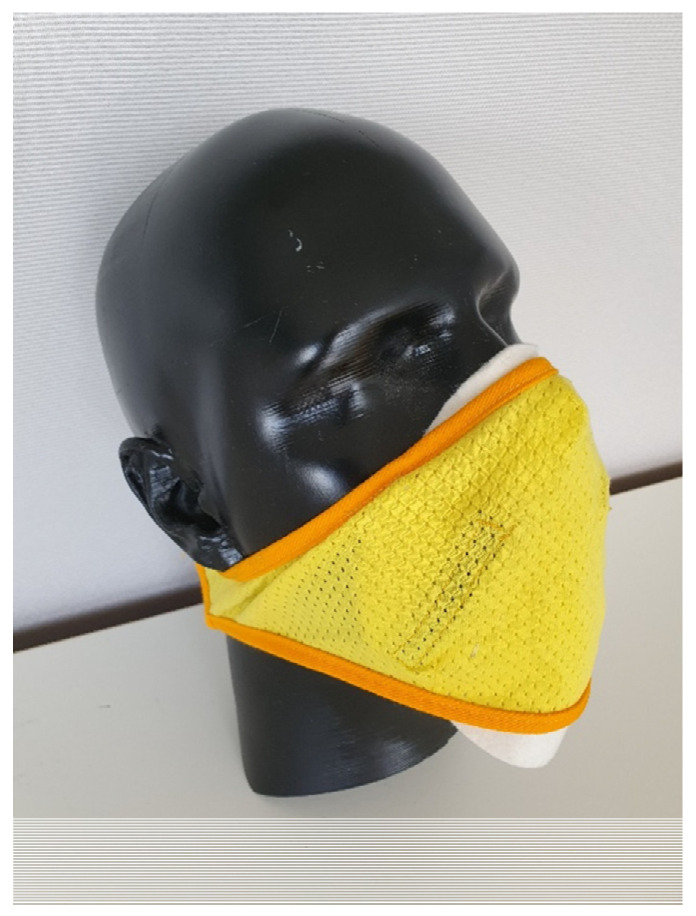
Wool–polypropylene fabric filter disc mounted with air permeable wrap to wearer’s face.

Fabric discs (160 mm diameter) were cut from the fabric and worn against the nose and mouth whilst held in place with a lightweight, air permeable warp knitted wrap (Fair Air Fire Mask without filter) secured at the back of the head by a hook and loop fastener (Figure 2).

This configuration, coupled with the low pressure drop of the filter media, ensured that air flow was predominately through the filter media as discussed below.

**Figure 3:**
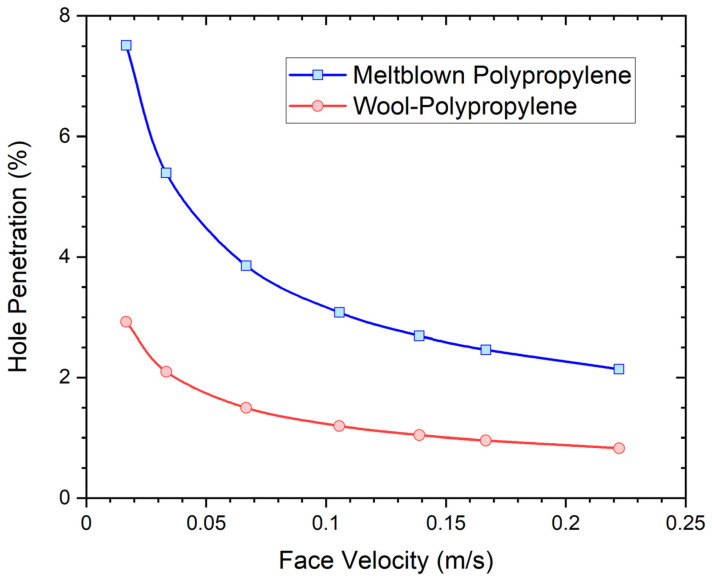
Modelled respirator leakage rates (hole penetration) as a function of face velocity for wool/polypropylene and melt blown polypropylene filter media with pressure drops of 30 Pa and 200 Pa at a face velocity of 0.15 m/s. Trendlines (modified Bézier) have been added for illustration purposes to calculated data represented by dots.

Commercially manufactured respirators are typically constructed with a perimeter designed for a good facial seal as high filtration efficiency is only possible when this is achieved. Ideally, respirator brand, type, and size should be fit tested to specific individuals before use to minimize potential leakage, and the respirator functions as intended. A small hole, poor fit, or even facial hair can significantly degrade performance. The fluid dynamics of air flow through respirators, with and without holes, can be satisfactorily modelled using both analytical and numerical models [21]. These models of air flow indicate that respirators with high pressure drop are more prone to side leakages. Given the significantly different pressure drop associated with the wool/polypropylene filter media (30 Pa) compared to typical, commercially available melt blown polypropylene respirators (typically approximately 200 Pa for a N95 respirator), the analytical model [21] was used to predict differences in potential leakage rates of an imperfectly fitted respirator. The modelled respirator was assumed to have an active area of 150 cm^2^, and pressure drops of 30 Pa and 200 Pa at a face velocity of 0.15 m/s for the wool/polypropylene and melt blown polypropylene filter media, respectively. An imperfect facial seal was represented by a hole with a square aperture of 2 mm sides and 2 mm length. The share of the airflow through the hole or leak relative to total airflow, referred to in the following as “hole penetration”, was calculated as a function of air face velocities for the two different filter media as shown in Figure 3. The model assumes that aerosol particles pass through the hole unabated and contribute accordingly to the overall penetration of the mask. Face velocities of 0.05 m/s and 0.15 m/s represent low and moderate physiological exertion levels. Modelling clearly indicates that for the same hole size, leakage is greater at lower face velocities (low physiological exertion) and is significantly higher for the filter medium with higher pressure drop or flow resistance (melt blown polypropylene). These trends have been verified experimentally across various respirator types.

### 2.3. Filtration Testing

Particle penetration and pressure drop were measured after samples were conditioned overnight at 65% relative humidity (r.h.) and 20 °C. The central 113 mm diameter of the sample (equivalent to 100 cm^2^ active area) was exposed to the challenge aerosol when located in the sample holder of the instrument [5]. A challenge aerosol of KCl was generated from a salt solution (10 g/L) with air at a face velocity of 0.15 m/s. The challenge aerosol had a median particle size of 0.6 µm and particle count concentration of 5 × 10^7^ per m^3^. Particles upstream and downstream of the filter sample were measured by an optical particle counter/sizer (TSI Aerotrak 9306-03) by taking a 2.8 L/minute sample from the airflow stream for the particle counter. Particle counts were measured across six size ranges over the total particle distribution range of 0.3–25 µm diameter, but the analysis focused on the most penetrating particles of 0.3–0.5 µm diameter (0.3 μm size range). For each test, five sequential measurements of particle counts were determined over a sampling period of 30 s. The effectiveness of the filter in removing particles was expressed as the penetration of particles in the 0.3 μm size range, (P_0.3_), the mean of the five measurements for the ratio of the number of particles measured downstream to those measured upstream. The penetration evaluated in this way is about four times higher than the penetration obtained from total particulate mass measurement, which is the method used by the Australian standard for respirator performance testing [11]. The highlighted green area in Figure 1 depicts the detection range of P_0.3_ and shows that wool–polypropylene and melt blown filter media have very similar sensitivities to loss of electrostatic enhancement in this range, while differences become much more pronounced for coarse particles and hence for total particulate mass. This distinct property of P_0.3_ allows representative comparisons between these media to be made. The pressure drop was also determined during testing. Testing was carried out after an initial heat (sanitization) treatment, then after wear and the second heat treatment.

The behavior of the filter media under a high concentration (45 mg/m^3^) methylene blue aerosol challenge [20] was determined as an indication of dust holding capacity.

### 2.4. Statistical Analysis

Mixed models for the logarithm of penetration (P_0.3_) were fitted with the lme4 package in R [22] and compared using analysis of variance to test the effects of areal density, heat treatment and wear time. Coefficients of determination *R*^2^ were calculated using the MuMIn package in R [23]. Penetration (P_0.3_) estimates and confidence intervals were calculated from 10,001 bootstrap samples.

## 3. Results

**Figure 4:**
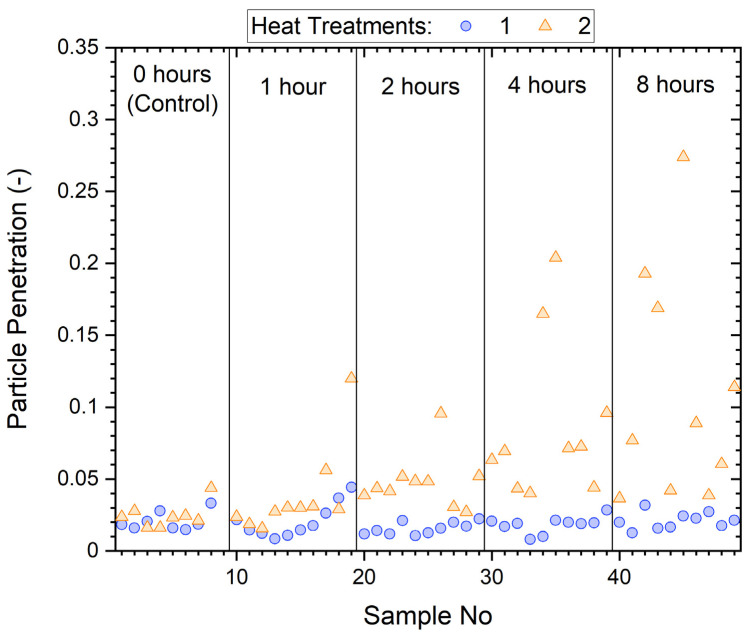
Penetration (P_0.3_) of mask samples before (circle) and after (triangle) wearing for specified times (1, 2, 4, and 8 h) and control samples that were not worn.

The particle penetrations, P_0.3_ (mean of five measurements/sample) of all samples before and after wear are shown in Figure 4. As noted above, we have found that P_0.3_ penetrations are typically four times higher than penetrations obtained from total mass measurement, a technique used in the study of worn N95 masks [13], and more sensitive to filtration performance deterioration. This is because the effect of loss of electrostatic charge for melt blown filter media (Figure 1) is much stronger in the sub-micrometer region than for multi-micrometer size coarse particles, where wool–polypropylene based filter media are most susceptible.

Before wear, samples had a penetration of ~2%, but after wear, penetration increased substantially for some samples (>10%), while others showed little change.

**Figure 5:**
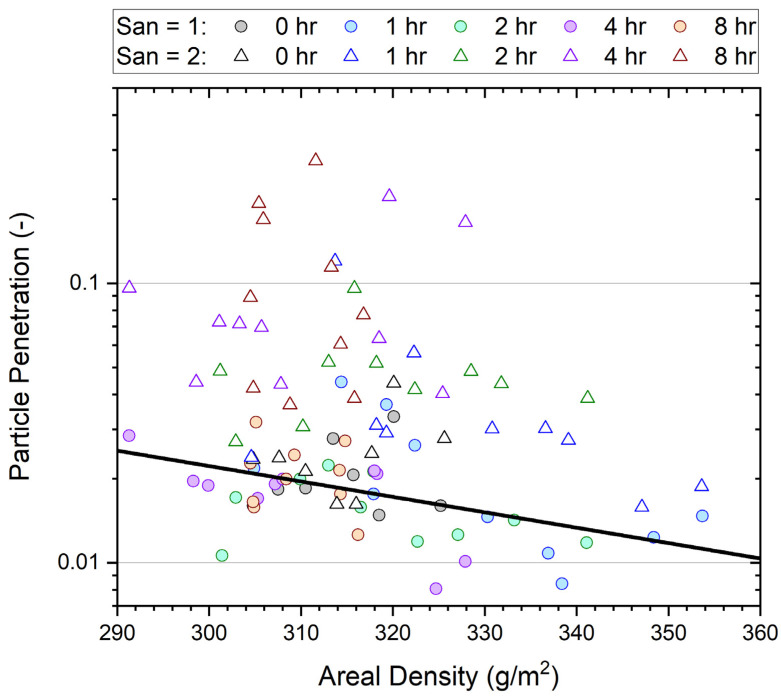
Penetration (P_0.3_) of samples before and after wearing for 0–8 h as a function of areal density (basis weight). Filter tests were conducted after the first (San = 1) and second (San = 2) heat sanitization treatments. A linear fit of the logarithm of the average particle penetration Log(P) over areal density for unworn media (San = 1) is shown to illustrate the expected trend.

Figure 5 plots all P_0.3_ data from the wear trial delineated based on wear and heat (sanitization) treatment (point shape) and time of wear (point color) against the areal density for each individual sample. Data in Figure 5 are in paired sets: circles are used to plot measurements of samples after one heat treatment, while triangles show measurements after either wearing or the control (two heat treatments). The areal density is essentially unchanged in each pair.

**Figure 6:**
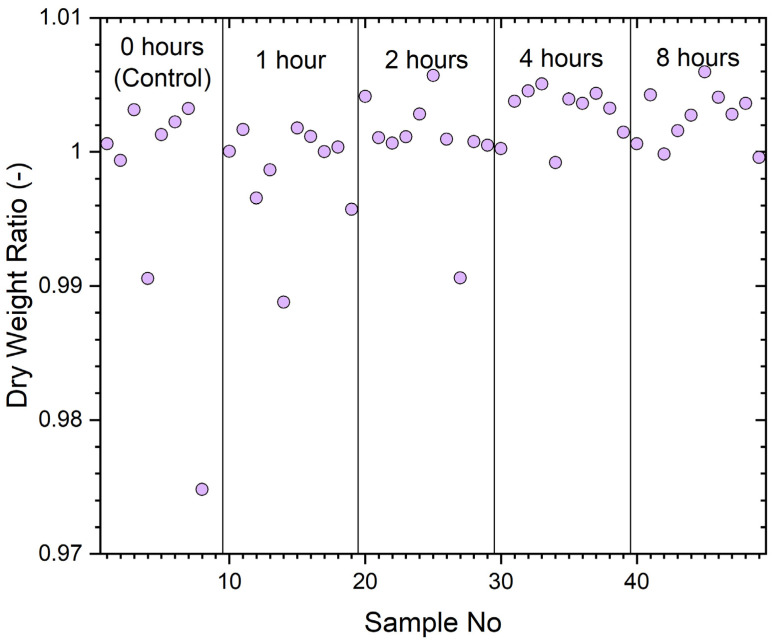
Dry weight ratio, worn/unworn of trial samples with wear times of 0, 1, 2, 4, or 8 h.

The dry weight ratios (worn/unworn) for each sample (including 0 h for unworn controls) are given in Figure 6. These weights were measured immediately after filter samples were removed from the oven after the sanitizing treatment.

The vertical lines delineate the period of wear. The typical ratio between 0.995 and 1.005 would be well inside the precision of the measurement technique as wool is very hydroscopic and samples rapidly gain moisture during weighing after they are removed from the desiccator. The results indicate that little dust (particulates) or nonvolatile human excretions are accumulated (less than ~50 mg) during the wear periods. The ratio of sample 8, a control sample, appears atypical.

**Figure 7:**
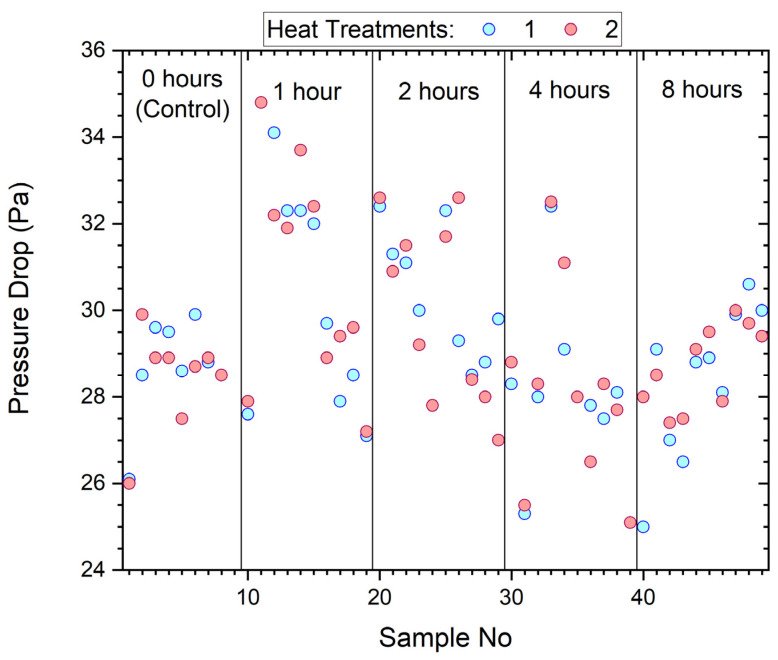
Pressure drop of samples before and after wearing by participants.

The pressure drop for samples measured before and after wearing and sanitization is shown in Figure 7. Also included are the control samples that were sanitized but not worn, shown between the narrowly spaced vertical lines.

The pressure drop of the samples ranged from 25–35 Pa with wear contributing little or no practical change.

**Figure 8:**
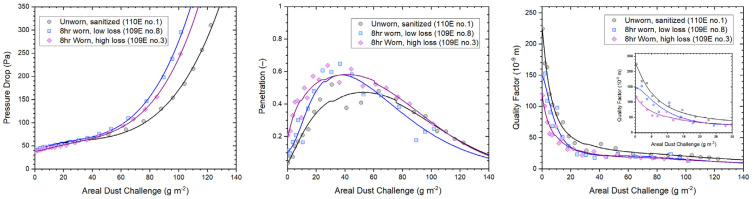
Dust loading performances of sanitized mask media in unworn and worn state. Low and high loss refer to the observed level of deterioration that the mask media incurred during wear. Mathematical fit functions used to illustrate dust loading dependencies were described in [25].

The WP medium is more reliant on electrostatic charge for small particle capture with higher penetration on charge depletion before performance starts to improve due to clogging and a slower increase in pressure drop compared to melt blown polypropylene [24]. The capacity of a mask medium to hold aerosol particles was assessed using a high concentration challenge of methylene blue aerosol in a dust loading performance test [7]. The performances of two mask media that were exposed to 8 h of wear, i.e., the longest exposure time of this investigation, are compared in Figure 8 to the performance of a sanitized, unworn mask medium with similar initial pressure drop. Parameters measured include changes with increasing dust load for pressure drop, penetration and quality factor. Based on a filter test area of 87 mm diameter (60 cm^2^), an area dust load challenge of 10 g/m^2^ corresponds to a dust load of approximately 60 mg. Note that as penetration increases and then decreases, the filter medium starts to clog up.

The quality factor is an indicator for filtration performance that combines pressure drop, penetration, and face velocity. Results in Figure 8 show clearly how performances of the three media deteriorate with increasing areal dust challenge, with the unworn medium starting at the highest performance level, followed by the medium that experienced low loss, and, finally, the medium with high loss.

## 4. Discussion

This investigation aimed to quantify how much and how fast the level of protection afforded by disposable respirators can deteriorate during wear in a live user setting. There are many parameters and conditions that can influence results, such as the charge that aerosol particles carry [26,27], their physical state (solid or liquid) [28,29] and the distribution of particle sizes [17], humidity and temperature [24,30], the type of electrostatic enhancement [6], active area, and design of the respirator mask [31], as well as environmental factors like the presence of ambient contaminants (e.g., vapors from disinfectants), donning and doffing practices, and mask reuse.

While results from this study are specific to the conditions under which experiments were conducted, the general findings are more widely applicable since properties of aerosols, electrostatically enhanced filter media, and consideration for mask designs all follow the same fundamental relationships and principles.

Results show that the main effect of wear was imposed on filtration penetration and dust holding capacity, while there is no clear effect or trend in changes to dry mass or pressure drop.

A general decrease in penetration as areal density increases is apparent in Figure 5. The relationship between pressure drop and the natural logarithm of penetration, ln(P), is linear, according to fundamental principles described by the ‘quality factor’ for filtration [32], with pressure drop increasing proportionally to areal density (basis weight). The data indicate a 1 g/m^2^ increase in areal density leads to a 0.95% reduction in penetration.

Penetration was significantly higher for higher particle counts in the test aerosol, though increasing the number of particles in the 0.3 µm size range for a constant total aerosol count leads to a decrease in the proportion of these particles that penetrate the mask (*p* < 0.0001). There is, thus, an instrument effect alongside the concentration related stress effect of the challenge aerosol on the filter medium. Quantifying the aerosol effect is important in order to allow unbiased comparisons between masks subjected to slightly different aerosols. Penetration tends to be slightly higher after the second heat treatment (*p* < 0.0001); the model suggests an increase of 20% on average. Penetration is clearly higher after wearing, compared to the unworn control, and the higher values are for longer hours of wear (*p* < 0.0001). The time effect is investigated in more detail below.

A model for average ln(P) that is based on an ansatz with linear and quadratic terms comprising the main influencing factors identified is, thus,

$$ln\left(P\right)=-0.86+0.066\;C_{tot}-0.15\;C_{0.3}+0.00057\;C_{0.3}^{2}-0.0095\;A+0.18\;s+0.45\;t-0.035\;t^{2}$$

Here the *C* coefficients give the total test aerosol particle concentration *C_tot_* (in particles per cm^3^) and the concentration for the 0.3 µm diameter size range *C*_0.3_; *A* is the areal density in g/m^2^; *s* is the number of sanitizations (1 or 2); and *t* is the number of hours worn.

For identical settings of areal density and aerosol concentration with two sanitization treatments, this reduces to a quadratic equation in the time for which the mask is worn, *ln(P)* = *k* + 0.45 *t* − 0.035 *t*^2^, where *k* is the natural logarithm of the initial penetration.

Figure 4 shows considerable variation between masks worn for the same length of time. The variance appears to increase for higher wear times. This effect is also included in the model. Given these random effects, the model has a fairly good marginal value of *R*^2^ of 68.5%.

**Figure 9:**
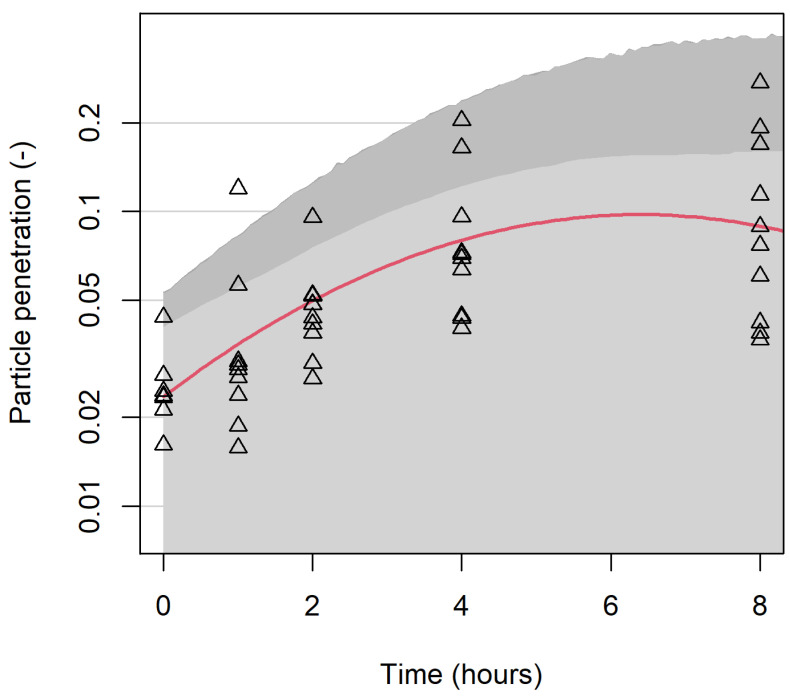
Penetration (P_0.3_) of samples after wearing for 0–8 h. Experimental results are shown by triangles; the red curve shows modelled mean penetration. Light shading gives 95% confidence intervals for mean penetration, and dark shading for observations on individual masks.

Figure 9 indicates the effects of wear time for samples with a nominal areal density of 320 g/m^2^, with a standard aerosol of 42 particles per cm^3^, half the particles being in the 0.3 µm size range. The lighter shaded area gives one-sided 95% confidence intervals for the mean penetration at each time and the darker shaded area gives a confidence interval for individual observations, allowing for random variation between masks. Observed data for all masks after the second sanitization are plotted for comparison. This figure demonstrates the physical meaning of the coefficients in *t* in the model: particle penetration increases markedly over the first four hours of wear, but was not observed to have worsened further over the next four hours.

Wear degrades respirator performance. The model was fitted on a log scale, which allows for increasing variance for higher wear times (as shown in Figure 4), leading to widening confidence intervals. This change is likely due to a combination of exhaled moisture (vapor and aerosolized) and non-volatile excretions as well as particles captured during inhalation, with the relative significance of each difficult to determine. The shape of the curve in Figure 9 could have a similar explanation to the shapes of the curves in the middle panel of Figure 8, which is that the pores in the filter medium of the mask could eventually become clogged. However, more data with longer exposure times would be needed to check that penetration does eventually decrease.

While the 5% penetration limit set by N95 and other test standards for respiratory protective devices (RPD) is useful to arrive at a pass/fail decision, it does not mean that people are at risk if the penetration is higher or safe otherwise. If facial seal is taken into account, the penetration can easily reach 10% or more, as reported in [14]. The protection afforded by these RPD is, nevertheless, highly significant and important for implementing an effective disease prevention control scheme.

## 5. Conclusions

The wear trial has demonstrated the wool/polypropylene electrostatic filter media with an areal density (basis weight) of ~320 g/m^2^ can be effective in limiting the most penetrating particles in the size range of ~0.3 μm to less than 5%.

The modelled data show increasing areal density by 1 g/m^2^ reduces penetration by 0.95%, identify a 20% increase in penetration attributable to the heat sanitization treatment itself, and quantify the effects of the test aerosol used in order to avoid bias in comparisons between masks. Considerable variation in filtration efficiency was also observed between different masks.

Importantly, penetration increases during wear; the longer the wear time, the more deleterious to particle removal, particularly after approximately 2 h of wear. This study provides a step towards understanding how much and how quickly the level of protection afforded by disposable respirators can deteriorate during wear in a live user setting.

## Figures and Tables

**Table 1:** Filter material description.

Property	Wool	Polypropylene	Processed Blend
Blend Composition (%)	60	40	-
Diameter (μm)	21.0	22.9	-
Length (mm)	43	67	-
Short Fiber content (%)	37	1	-
Fiber Type	Carbonized crutchings and locks	3 denier staple fiber with hydrophilic finish	-
Cleaning	6 bowl machine-scouring in 65 °C water, BD40 detergent in bowls 1 and 2		-
Areal Density (g/m^2^)	-		≈320
Pressure Drop (Pa) at face velocity of 0.15 m/s	-		≈30
Penetration P_0.3_ (%), unworn (%)	-		≈2

## Data Availability

The data presented in this study are openly available from the CSIRO Data Access Portal (DAP) at https://doi.org/10.25919/qsjq-p934 (accessed on 14 April 2022).
